# Short-Term Heart Rate Variability Dynamics and Mortality Risk After Acute Coronary Syndrome

**DOI:** 10.3390/diagnostics16060942

**Published:** 2026-03-23

**Authors:** Nikola Marković, Maša Petrović, Silvana Babić, Milovan Bojić, Branislav Milovanović

**Affiliations:** 1 Institute for Cardiovascular Diseases “Dedinje”, 11000 Belgrade, Serbia; 2 Clinic for Otorhinolaryngology and Maxillofacial Surgery, University Clinical Center of Serbia, 11000 Belgrade, Serbia; 3 Faculty of Medicine, University of Banja Luka, 78000 Banja Luka, Bosnia and Herzegovina; 4 School of Medicine, University of Belgrade, 11000 Belgrade, Serbia

**Keywords:** heart rate variability, acute coronary syndrome, autonomic nervous system, short-term HRV, electrocardiology

## Abstract

**Background/Objectives:** Heart rate variability (HRV) is a non-invasive marker of autonomic nervous system function with established prognostic value after acute coronary syndrome (ACS). The clinical relevance of temporal changes in short-term HRV remains insufficiently defined. This study evaluated short-term HRV dynamics and their association with mortality after ACS. **Methods:** This retrospective–prospective study included 230 patients with acute myocardial infarction. Five-minute resting ECG recordings were obtained on day 1 and day 21. Time- and frequency-domain HRV parameters were analyzed, and delta values were calculated. The primary endpoint was overall mortality. Survival was assessed using Kaplan–Meier analysis and Cox regression. **Results:** Patients who died during follow-up had lower HRV values on day 21 and more pronounced declines in selected parameters. In multivariable analysis, decreased ΔLF and shorter RR intervals independently predicted overall mortality. **Conclusions:** Short-term HRV provides a practical bedside assessment of autonomic function after ACS. Unfavorable temporal changes likely reflect persistent autonomic imbalance and may offer additional prognostic insight. Larger contemporary studies are needed to confirm these findings.

## 1. Introduction

The autonomic nervous system (ANS) constitutes a complex neural network that plays a central role in regulating physiological responses to internal and external stressors, thereby facilitating adaptation and maintaining systemic homeostasis [1]. Consequently, disturbances in ANS function are implicated in a broad spectrum of pathological conditions, contributing not only to disease development but also to clinical presentation and long-term prognosis [2,3,4,5,6].

Numerous methods are available for the assessment of ANS function, including cardiovascular reflex testing, electrocardiographic analysis, heart rate analysis, baroreflex sensitivity testing, and skin biopsy–based evaluation of epidermal nerve fiber density and sudomotor gland nerve fiber density (ENFD and SGNFD) [7,8,9,10,11]. Among these approaches, heart rate variability (HRV) analysis remains one of the most widely applied and clinically accessible tools [7,8].

HRV has been extensively investigated as a risk stratification marker following acute coronary syndrome (ACS), as well as in patients with heart failure (HF), in whom reduced values of specific HRV parameters have been associated with adverse outcomes [12,13]. In the context of ACS, most studies have relied on 24-h Holter electrocardiogram (ECG) recordings obtained at a defined time point after the acute event—typically between day 1 and day 28 or following hospital discharge—thus primarily reflecting long-term HRV assessment [12,14,15,16,17,18,19,20,21,22,23,24].

In contrast, short-term HRV analysis does not necessarily require 24-h Holter ECG monitoring (unless specific intervals are selected), is more feasible for bedside assessment, and is typically derived from recordings of approximately 5 minutes’ duration, although shorter protocols have also been described [7,8]. Importantly, the physiological underpinnings and the relative contributions of sympathetic and parasympathetic modulation differ between short- and long-term recordings [8]. Previous investigations employing short-term or ultra-short-term HRV (e.g., 10-s recordings) have demonstrated reduced parameter values under certain conditions and suggested potential prognostic significance for mortality after ACS [25,26,27,28,29,30].

A distinctive feature of autonomic remodeling following ACS is the development of morphological and structural changes collectively referred to as ischemic denervation [31]. This process persists even after revascularization and optimal medical therapy. It is driven by altered afferent signaling and further modulated by cytokine-mediated inflammatory responses. The resulting changes include sustained sympathetic activation accompanied by structural and neurochemical remodeling, such as stellate ganglion remodeling, hyperinnervation of infarct border zones, neuronal hypertrophy, and reduced afferent input from the infarcted myocardium [32,33,34,35,36,37,38].

Despite these dynamic neuroanatomical and neurochemical alterations, most previous HRV studies—though not all—have relied on measurements obtained at a single time point, thereby failing to capture temporal changes in ANS function during the subacute recovery period after ACS.

The aims of this study are (1) to evaluate short-term HRV recorded on day 1 and day 21 after ACS and compare HRV parameters and their temporal changes between survivors and non-survivors during follow-up, and (2) to identify HRV-derived variables associated with overall mortality and evaluate their prognostic value.

## 2. Materials and Methods

### 2.1. Study Protocol

This study was designed as a retrospective cohort with prospective follow-up and included 230 patients admitted between 2003 and 2013 to the Coronary Care Unit of the Clinical Hospital Center Bežanijska Kosa in Belgrade, Serbia, with a diagnosis of acute myocardial infarction (AMI). Of the total study population, 35 patients died during follow-up, while 195 survived. Initial management consisted of fibrinolytic therapy or conservative treatment, primarily determined by time from symptom onset and/or contraindications to fibrinolytic therapy [39,40]. All patients received standard-of-care medical therapy in accordance with contemporary clinical guidelines.

Exclusion criteria included terminal-stage malignancy, severe respiratory or renal failure, and other severe comorbid conditions limiting reliable assessment. Only patients in sinus rhythm were included in the analysis; therefore, individuals with persistent atrial fibrillation or atrial flutter, pacemaker rhythm, or any other non-sinus rhythm at the time of ECG recording were excluded. Patients who were hemodynamically unstable at the time of assessment, as well as cases with incomplete or inaccurate data, were also excluded.

The study adhered to the principles of the Declaration of Helsinki and received initial approval from the Scientific Ethical Committee of the University Clinical Center “Bežanijska Kosa” (protocol code 1039/3; approval date 12 April 2011). It was subsequently re-approved by the Ethics Committee of the Institute for Cardiovascular Diseases “Dedinje” (protocol code 6470; re-approval date 11 December 2024). The study was supported by grant TP 32040 from the Ministry of Education, Science, and Technological Development of the Republic of Serbia.

Data were extracted from the medical records of all eligible patients. Collected information included clinical characteristics at the time of admission, relevant diagnostic tests, and imaging performed during hospitalization. The analyzed variables included: (1) demographic data (age and gender), (2) in-hospital pharmacotherapy, (3) medical history of previous cardiovascular events, (4) cardiovascular risk factors, (5) infarct localization (anteroseptal (AS), inferoposterior (IP), non-ST elevation MI (NSTEMI), and other localizations (e.g., right ventricular or lateral infarction)), (6) clinical status at admission, (7) echocardiographic parameters assessed within 24 h after admission, and (8) short-term ECG recordings used for HRV analysis obtained on day 1 and day 21 after admission.

Short-term ECG recordings were obtained using a standard 12-lead electrocardiograph (Schiller AG, Baar, Switzerland) during resting conditions and recorded for 5 min. Recordings were performed on day 1 and day 21 following admission. Participants were instructed to maintain calm, spontaneous breathing and to avoid deep or irregular respiratory patterns in order to minimize respiration-related variability in spectral HRV measures. All recordings were conducted during morning hours.

The following ECG parameters were analyzed: P wave duration, PQ interval, QRS duration, QTc interval (calculated using Bazett’s formula), and RR interval length. HRV analysis included time-domain parameters—standard deviation of NN intervals (SDNN), percentage of successive NN intervals differing by more than 50 ms (pNN50), and root mean square of successive differences (RMSSD)—as well as frequency-domain parameters, including very-low-frequency (VLF), low-frequency (LF), and high-frequency (HF) components, and the LF/HF ratio. HRV parameters were calculated using the commercial software integrated within the electrocardiograph (Schiller AG, Baar, Switzerland). The software performs automated RR interval detection and HRV analysis in accordance with previously established standards proposed by the Task Force of the European Society of Cardiology and the North American Society of Pacing and Electrophysiology [8]. Signal preprocessing, including artifact detection, ectopic beat identification, and exclusion of noisy segments, was performed automatically by the software according to predefined internal algorithms. For all ECG and HRV parameters, delta (Δ) values were calculated as the difference between measurements obtained on day 21 and day 1.

Following hospital discharge, patients were monitored for clinical outcomes of interest, with overall mortality defined as the primary endpoint. Follow-up data was obtained from medical records and structured telephone interviews to determine survival status after the index hospitalization. After a median follow-up of 46 months (interquartile range: 27–60 months), 35 patients (15.2%) had died from all causes.

### 2.2. Statistical Analysis

Results are presented as mean and standard deviation (SD), median (Mdn) with 25–75% interquartile range (IQR), or counts and percentages, depending on the data type. Continuous variables were tested for normality using the Kolmogorov–Smirnov test, as well as visual inspection of normal and detrended normal Q–Q plots. Group comparisons were performed using parametric and nonparametric tests, as appropriate. Normally distributed continuous variables were compared using the independent-samples *t*-test, whereas non-normally distributed variables were analyzed using the Mann–Whitney U test. Categorical variables were compared using the Chi-square test or Fisher’s exact test, as appropriate. The level of statistical significance was set at *p* < 0.05.

Kaplan–Meier survival analysis was used to estimate survival probability, with overall mortality defined as the primary endpoint. Survival time was calculated from the index hospitalization until death from any cause or the end of follow-up. Continuous variables were categorized using median values or pre-established cutoff points. Delta values were categorized according to the direction of change (increase vs. decrease). A small number of patients showed identical values at both time points; due to the absence of events in this subgroup and limitations for regression modeling, these cases were assigned to the “increase” category. To examine associations between predictors and time-to-event outcomes, univariable Cox proportional hazards regression analysis was performed. Hazard ratios (HRs) with 95% confidence intervals (CIs) were calculated for each variable. All variables reported in the Results section were systematically evaluated in the univariable Cox proportional hazards regression analysis. Variables with *p* < 0.05 in univariable analysis were considered for entry into the multivariable model. For variables available in both continuous and categorical forms, continuous versions were retained in the final model due to their greater discriminatory capacity. Multivariable Cox regression analysis was performed using a forward conditional stepwise selection procedure. In this approach, all candidate predictors identified in the univariable analysis were initially considered for entry into the multivariable model. At each step, variables not yet included in the model were evaluated using the score statistic, and the variable with the lowest *p* value below the predefined entry threshold (*p* < 0.05) was entered into the model. After a variable was entered, all remaining candidate predictors were again evaluated to determine whether their inclusion would significantly improve model fit. Variables were retained in the model only if their statistical significance remained below the predefined retention threshold (*p* < 0.10). The selection procedure continued step by step until no additional variables met the entry criterion. Due to the nature of the forward conditional stepwise procedure, hazard ratios and corresponding *p* values are presented only for variables retained in the final multivariable model. Variables that were evaluated during the stepwise selection process but did not meet the predefined entry criterion were therefore not included in the final model.

All statistical analyses were performed using SPSS version 26.0 (IBM Corp., Armonk, NY, USA).

## 3. Results

Baseline characteristics and clinical status upon admission are presented in Table 1. Patients who died during follow-up were significantly older than survivors (62.3 ± 9.8 vs. 58.1 ± 8.8 years, *p* = 0.009). They also more frequently presented with higher Killip classes (*p* = 0.017) and atrioventricular (AV) block grade II–III (11.4% vs. 3.1%, *p* = 0.048).

No other baseline demographic variables, cardiovascular risk factors, or admission clinical characteristics differed significantly between groups.

Echocardiographic parameters are summarized in Supplementary Table S1. The mean left ventricular ejection fraction (LVEF) was approximately 45% in both groups, and 30–40% of patients in each group had LVEF ≤ 40%, without statistically significant differences.

Pericardial effusion was more frequently observed among patients who died during follow-up (17.1% vs. 5.6%, *p* = 0.028).

In-hospital medical therapy is shown in Supplementary Table S2. Survivors were more frequently treated with beta-blockers (79% vs. 62.9%, *p* = 0.038) and unfractionated or low-molecular-weight heparin (UFH/LMWH) (91.3% vs. 77.1%, *p* = 0.033).

No other significant differences in medical therapy were observed. Approximately 20% of patients in both groups received fibrinolytic therapy, and nearly 90% received dual antiplatelet therapy (DAPT).

Conventional ECG parameters at day 1 and day 21, as well as corresponding delta (Δ) values, are presented in Supplementary Table S3. No statistically significant differences were observed between groups.

Although median ΔQRS did not change in patients who died, the QRS duration tended to increase in survivors; however, this difference did not reach statistical significance.

Short-term time-domain parameters of HRV at Day 1 and Day 21 after myocardial infarction and corresponding delta values of the study population are shown in Table 2. The values of the parameters on the 1st day did not differ significantly. On the other hand, patients who died at the end of follow-up had significantly lower values of parameters at the 21st day. When temporal changes were analyzed, both SDNN and RMSSD decreased from day 1 to day 21 in deceased patients relative to survivors; however, only ΔSDNN reached statistical significance (*p* = 0.012). ΔRMSSD demonstrated a borderline association (*p* = 0.053).

Short-term frequency-domain parameters of HRV at Day 1 and Day 21 after myocardial infarction and corresponding delta values of the study population are shown in Table 3. No statistically significant differences regarding the values of the parameters on the 1st day were observed between groups. On the 21st day, patients who died had statistically lower values of all frequency-domain parameters, except LF/HF. Regarding temporal changes, ΔLF (*p* = 0.020) and ΔHF (*p* = 0.012) were significantly lower in patients who died.

**Table 3:** Short-term frequency domain parameters of Heart Rate Variability at Day 1 and Day 21 after myocardial infarction and corresponding delta values of the study population.

	Died *N* = 35	Survived *N* = 195	*p* Value
**1st day**			
VLF (ms^2^) (Mdn (IQR))	51 (20–163)	76 (30–179)	0.221 ^m^
LF (ms^2^) (Mdn (IQR))	57 (17–163)	57 (17–124)	0.777 ^m^
HF (ms^2^) (Mdn (IQR))	23 (9—63)	21 (7–93)	0.957 ^m^
LF/HF (Mdn (IQR))	2.7 (0.8–5.5)	2 (1.1–4.9)	0.789 ^m^
**21st day**			
VLF (ms^2^) (Mdn (IQR))	44 (14–111)	68 (34–149)	0.036 ^m^
LF (ms^2^) (Mdn (IQR))	24 (6–49)	42 (16–96)	0.009 ^m^
HF (ms^2^) (Mdn (IQR))	10 (3–26)	23 (9–63)	0.001 ^m^
LF/HF (Mdn (IQR))	2 (1.1–5.1)	1.7 (0.8–3.3)	0.145 ^m^
Δvalues (after–before) *			
VLF (ms^2^) (Mdn (IQR))	−4 (−90–32)	1 (−96–54)	0.422 ^m^
LF (ms^2^) (Mdn (IQR))	−22 (−104–0)	−4 (−60–29)	0.020 ^m^
HF (ms^2^) (Mdn (IQR))	−11 (−53–0)	0.1 (−34–26)	0.012 ^m^
LF/HF (Mdn (IQR))	−0.2 (−2.9–1.2)	−0.4 (−2.2—0.7)	0.925 ^m^

VLF—Very Low Frequency; LF—Low Frequency; HF—High Frequency; ms^2^—milliseconds squared; *—Δ values represent change from baseline (after–before); Mdn—Median; IQR—Interquartile range (25–75%); ^m^—Mann–Whitney U test.

All significant predictors of mortality after ACS in the univariable and final multivariable Cox Analysis model are presented in Table 4. In the final model, age was a positive independent predictor of overall mortality, as well as decreased values of ΔLF, with a Hazard ratio of 2.3. On the other hand, length of RR interval was inversely associated with overall mortality.

Supplementary Tables S4–S6 present baseline characteristics, echocardiographic parameters, and in-hospital therapy stratified according to ΔLF categories. The only significant difference observed was a higher prevalence of hypertension among patients with increased ΔLF compared with those with decreased ΔLF (78.4% vs. 64.7%, *p* = 0.025).

**Figure 1:**
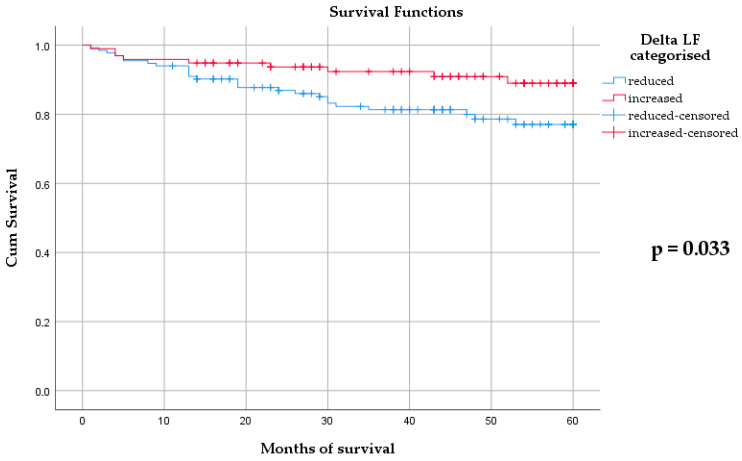
Kaplan–Meier curves for overall mortality stratified by ΔLF categories. Log-rank *p* = 0.033. Number at risk at months 0, 20, 40, and 60: ΔLF decrease = 133, 109, 78, 0; ΔLF increase = 97, 82, 64, 0.

Kaplan–Meier survival curves stratified by ΔLF categories are presented in Figure 1. Patients with decreased ΔLF demonstrated significantly worse overall survival compared with those with increased ΔLF (log-rank *p* = 0.033).

Detailed results of the univariable Cox regression analyses for all examined variables are provided in Supplementary Tables S7–S12.

Supplementary Table S13 shows the results of additional linear and logistic regression analyses assessing the association between beta-blocker therapy and changes in the LF component of HRV. Beta-blocker therapy was not significantly associated with ΔLF in either model.

## 4. Discussion

In this study, patients who died during follow-up exhibited significantly lower values of several short-term HRV parameters, primarily SDNN, VLF, LF, and HF, suggesting the presence of autonomic imbalance and relative parasympathetic withdrawal within the first three weeks after ACS. In addition, a reduction in LF, along with age and RR interval duration, emerged as a significant predictor of overall mortality. These findings highlight the potential importance of temporal changes in ANS function and support the use of short-term HRV as a tool for risk stratification after ACS.

As shown in Table 1 and Table 4, patients with fatal outcomes were older, and age independently predicted overall mortality. The demographic characteristics of our cohort are consistent with prior studies of acute MI, with a predominance of male patients and comparable age distribution [41,42]. These findings further support the well-established role of older age as an important determinant of adverse outcomes after myocardial infarction.

Higher Killip class and second- to third-degree AV block were more frequent among patients who died and were significant in the univariable model but did not remain independent predictors in the final model (Table 1 and Table 4). The Killip classification reflects heart failure severity at presentation and is a well-established predictor of mortality after ACS, incorporated into risk scores such as GRACE [43]. The absence of significance in the final model may be explained by the study population, as only two patients had true cardiogenic shock (Killip IV).

The prognostic relevance of AV block depends on infarct localization. In inferior STEMI, AV block is often transient, whereas complete AV block may reflect larger infarcts and impaired ventricular function [44]. In anterior MI, AV block typically indicates extensive septal involvement and is associated with worse outcomes [44]. In the present cohort, the limited number of cases may have attenuated its independent prognostic contribution.

With regard to echocardiographic findings, as shown in Supplementary Table S1, pericardial effusion was significantly more frequent in patients who died and emerged as a predictor in univariable analysis (Table 4). Previous studies have highlighted the importance of pericardial effusion, as well as its size, as a marker of high-risk populations, primarily reflecting susceptibility to mechanical complications (i.e., free wall rupture) [45]. Moreover, Figueras et al. demonstrated that even mild early pericardial effusion after STEMI may indicate a higher risk of severe complications, as it is associated with hemopericardium and an increased likelihood of cardiac tamponade and free wall rupture [46].

As shown in Supplementary Table S2, patients who died had lower rates of beta-blocker and UFH/LMWH use, with both emerging as significant predictors in the univariable model (Table 4). Beta-blockers remain among the most commonly used therapies in both the acute and chronic phases after myocardial infarction [47]. Evidence from earlier trials has demonstrated that beta blockers reduce reinfarction, ventricular arrhythmias, and mortality, although much of this data originates from the pre-reperfusion era [44]. Their benefits have subsequently been extrapolated across the spectrum of ACS and are most pronounced in patients with EF ≤ 40% [44,47]. Anticoagulant therapy with heparin, including UFH and LMWH, represents an important component of ACS management [47]. Earlier studies showed that UFH reduces reinfarction and thromboembolic complications, while LMWH further lowers the risk of infarct-related artery reocclusion, recurrent ischemic events, and short-term mortality [44,47].

The most notable findings were the observed differences in short-term HRV parameters. As shown in Table 2 and Table 3, there were no significant differences between the two groups on the first day. In contrast, almost SDNN, VLF, HF, and LF were significantly lower in the group of patients who died during follow-up. As previously mentioned, most earlier studies have relied on long-term HRV, whereas this study used short-term HRV, which enables shorter, more practical bedside recordings under real-time conditions and does not require extensive post-processing such as 24 h Holter ECG analysis. However, it is important to emphasize that physiological differences exist between short- and long-term HRV measures, and the same parameters cannot be interpreted interchangeably [8]. During a five-minute resting recording, HRV is predominantly influenced by parasympathetic activity, whereas 24 h recordings reflect exposure to various stressors and a greater contribution of the sympathetic component of the ANS [8].

SDNN itself, reflecting overall ANS activity, represents the gold standard for risk stratification in post-ACS patients when assessed over 24-h recordings, and its prognostic value has also been demonstrated in patients treated with percutaneous coronary intervention (PCI) and fibrinolytic therapy [8,48,49]. However, short-term SDNN has also shown clinical relevance, being significantly higher in patients treated with fibrinolytic therapy compared with conventional treatment [25]. Furthermore, Vaage et al. demonstrated that daytime SDNN < 30 ms and nighttime SDNN < 18 ms, derived from five-minute recordings obtained during day and night periods, were independent predictors of 9-year mortality after ACS [26].

As mentioned, in addition to SDNN, several frequency-domain parameters, including VLF, LF, and HF, also differed significantly between the two groups, with lower values observed in patients who died during follow-up (Table 3). It has been suggested that, in five-minute recordings, frequency-domain measures may provide more informative insights than time-domain indices [8]. When recorded over a five-minute period, VLF typically contains approximately 5–12 oscillatory cycles [8]. It is influenced by multiple physiological mechanisms, including thermoregulatory, renin–angiotensin, endothelial, and physical activity–related factors, with contributions from parasympathetic activity and intrinsic cardiac neural networks, while sympathetic activation primarily modulates its amplitude and frequency [8]. In the Framingham study, VLF was identified as a significant predictor of overall mortality and adverse outcomes in patients with heart failure, with its prognostic value thought to reflect cardiac and systemic stress [50,51,52].

On the other hand, HF is known to reflect parasympathetic nervous system activity and corresponds to heart rate variability during respiration (the “respiratory band”) [8]. Lower values observed in patients who died further support the presence of parasympathetic withdrawal as one of the autonomic alterations following ACS [32,33,34,35,36,37,38]. In contrast, LF, which was initially considered a marker of sympathetic activity, may reflect baroreceptor-mediated modulation during resting conditions as well as the combined influence of both sympathetic and parasympathetic components [7,8]. Short-term LF has also been shown to predict sudden cardiac death in patients with heart failure, as demonstrated by La Rovere et al. [53]. Reduced LF may reflect impaired sinus node responsiveness, loss of physiological oscillatory behavior due to chronic sympathetic overactivity, central autonomic dysregulation, or reduced baroreflex sensitivity [53]. Given that LF variability is partly vagally mediated, parasympathetic withdrawal likely also contributes to its reduction [8,53].

In addition to differences in absolute values at 21 days, Δvalues of SDNN, HF, and LF were significantly lower and showed a decreasing trend in patients who died during follow-up (Table 2 and Table 3). These changes may reflect the previously described dynamic morphological and functional alterations of the ANS after ACS, primarily characterized by sympathetic predominance and parasympathetic withdrawal [32,33,34,35,36,37,38]. Moreover, Vaage Nielsen et al. demonstrated sustained daytime sympathetic predominance after ACS, with partial nighttime recovery of parasympathetic activity, using short-term SDNN in predefined recording periods [26]. Similarly, Detollenaere et al., using cardiovascular reflex tests, verified autonomic imbalance both at 2 and 6 weeks after MI, characterized by a rapid but transient decrease in vagal activity and increased orthosympathetic tone [54].

Finally, as shown in Table 4, apart from age, the final model identified average RR interval length during the five-minute recording on the 21st day and decreased ΔLF (compared with increased values) as independent predictors of overall mortality (Figure 1). The length of RR intervals on the 21st day after ACS was negatively associated with mortality and represents the reciprocal of heart rate. Previous studies have highlighted the clinical relevance of heart rate as a simple and widely available marker for risk stratification, likely reflecting underlying autonomic nervous system activity [32,55]. In contrast, decreased ΔLF was strongly associated with overall mortality. In the absence of identifiable confounders (Supplementary Tables S4–S6), decreased ΔLF may reflect intrinsic autonomic dysregulation. Considering the physiological mechanisms underlying LF variability, this finding may indicate disturbed autonomic balance, potentially involving impaired baroreflex function, which has been recognized as an important prognostic marker after ACS in prior studies [8,56,57,58]. In summary, all these findings suggest that reductions and temporal changes in selected short-term HRV parameters may reflect persistent autonomic imbalance after ACS, particularly parasympathetic withdrawal. Given the simplicity and feasibility of short-term recordings, this approach may offer a practical means of assessing post-ACS autonomic dynamics and further risk stratification.

An important consideration when interpreting the present findings relates to the treatment strategies applied in the study population. Although PCI is currently regarded as the standard of care for acute MI, fibrinolytic and conservative approaches remain clinically relevant in specific clinical settings, particularly in cases of delayed presentation or limited access to invasive treatment [47,58,59]. Following myocardial infarction, autonomic dysfunction develops as part of a dynamic remodeling process characterized by evolving sympathetic–parasympathetic imbalance over time [32,33,34,35,36,37,38]. This process is continuous and involves ongoing neurochemical, structural, and signaling alterations within the cardiac autonomic network that persist even after successful treatment of the acute ischemic event [32,33,34,35,36,37,38]. Importantly, the prognostic value of HRV parameters has been demonstrated across different reperfusion strategies, including both PCI- and fibrinolysis-treated cohorts [48,49]. However, contemporary PCI-based management and advances in secondary prevention may influence their magnitude and temporal profile. Previous studies have also reported acute changes in HRV parameters associated with PCI. Increases in the LF/HF ratio accompanied by reductions in SDNN, RMSSD, LF, HF, TP, and SD2/SD1 have been observed following revascularization [30,60]. In some studies, HRV was assessed using short-term ECG recordings obtained before and approximately 24 h after PCI, while others evaluated HRV continuously during the procedure itself [30,60]. These findings suggest that PCI may induce immediate alterations in autonomic nervous system activity. In addition to procedural factors such as PCI, emerging evidence suggests that contemporary optimal pharmacological therapies may also influence autonomic function after myocardial infarction. In particular, Sodium Glucose Cotransporter 2 (SGLT2) inhibitors have been shown to improve HRV parameters and reduce sympathetic activity, accompanied by improvements in cardiac function and lower rates of adverse cardiovascular events [61]. These findings support the sympathoinhibitory effects of SGLT2 inhibitors and suggest a potential modulatory role in post-infarction autonomic remodeling [61]. In addition to SGLT2 inhibitors, mineralocorticoid receptor antagonists may also influence autonomic regulation after myocardial infarction. For example, administration of spironolactone has been shown to reduce cardiac sympathetic nerve activity and prevent left ventricular remodeling in patients with a first STEMI, highlighting its potential role in modulating post-infarction autonomic dysfunction, particularly in ischemic heart failure [62]. Furthermore, emerging data suggest that angiotensin receptor–neprilysin inhibitor therapy (sacubitril/valsartan) is also associated with favorable changes in cardiac autonomic indices, including modest increases in HRV and slight reductions in heart rate during the early months of treatment in patients with HFrEF. Therefore, both contemporary interventional strategies and evolving pharmacological therapies should be considered when interpreting autonomic alterations after myocardial infarction, and their potential impact on HRV parameters warrants further investigation in future studies.

### Study Limitations

Several limitations should be acknowledged. First, the relatively modest sample size limits the generalizability of the findings, and larger cohorts would be desirable to confirm the observed associations. Although the number of outcome events allowed multivariable analysis with an acceptable events-per-variable ratio in the final model (>10), a higher number of events would further strengthen the robustness of the results and might allow additional variables to emerge as independent predictors. Second, the categorization of certain variables, particularly ΔHRV parameters, may have influenced the findings. Dynamic changes were dichotomized into increased versus decreased values, while only a very small number of patients exhibited unchanged values. This approach, although pragmatic, may have affected the estimates, and alternative modeling strategies (e.g., continuous analyses or different categorization schemes) could potentially yield different results. Third, HRV was assessed at predefined timepoints (day 1 and day 21), and different recording intervals might capture alternative patterns of autonomic recovery or deterioration. Future studies incorporating multiple or continuous timepoints may provide a more comprehensive understanding of post-infarction autonomic dynamics. Another potential limitation relates to the influence of respiration on HRV parameters. Although patients were instructed to maintain calm, spontaneous breathing during the recordings, respiratory rate was not directly monitored or standardized. Since frequency-domain HRV indices, particularly the HF component, are known to be influenced by respiratory patterns, this may have introduced additional variability in the measurements. Fourth, the study lacked detailed coronary angiographic and procedural data, including culprit lesion characteristics, extent of coronary artery disease, and completeness of revascularization, all of which may influence prognosis and potentially interact with autonomic markers. Finally, the treatment context reflects the clinical setting in which the study was conducted. As contemporary MI management increasingly relies on PCI-based strategies, the present findings should be validated in larger and more contemporary cohorts.

## 5. Conclusions

Heart rate variability represents a well-established non-invasive method for assessing ANS function, applicable in both long- and short-term recordings. In this study, short-term HRV analysis identified significant differences in autonomic dynamics during the early post-infarction period, with reduced values and unfavorable temporal trends of selected parameters observed in patients who died during follow-up. These temporal changes likely reflect persistent autonomic imbalance characterized by sympathetic predominance, parasympathetic withdrawal and possible impairment of baroreflex function. Importantly, decreased ΔLF and shorter RR interval duration emerged as independent predictors of overall mortality, suggesting that dynamic autonomic changes could carry prognostic relevance after acute myocardial infarction. Given the feasibility, simplicity, and bedside applicability of five-minute ECG recordings, short-term HRV assessment may offer a practical electrocardiology-based approach for evaluating post-ACS autonomic remodeling and improving risk stratification. Nevertheless, these findings should be interpreted cautiously and validated in larger, contemporary cohorts treated predominantly with modern PCI-based strategies.

## Figures and Tables

**Table 1:** Baseline characteristics and clinical status upon admission of the study population.

	Died *N* = 35	Survived *N* = 195	*p* Value
**Baseline characteristics**			
Age (yrs.) (mean ± SD)	62.3 ± 9.8	58.1 ± 8.8	0.009 ^t^
Male (*n*, %)	21 (60%)	132 (67.7%)	0.375 ^c^
Previous MI (*n*, %)	4 (11.4%)	17 (8.7%)	0.536 ^f^
Hypertension (*n*, %)	23 (65.7%)	139 (71.3%)	0.506 ^c^
Hyperlipoproteinemia (*n*, %)	19 (54.3%)	133 (68.2%)	0.109 ^c^
Active smoking (*n*, %)	17 (48.6%)	118 (60.5%)	0.186 ^c^
Family history of CVD (*n*, %)	13 (37.1%)	98 (50.3%)	0.153 ^c^
Diabetes Mellitus (*n*, %)	16 (45.7%)	66 (33.8%)	0.177 ^c^
**Clinical status upon admission**			
AS MI (*n*, %)	10 (28.6%)	55 (28.2%)	0.717 ^c^
IP MI (*n*, %)	16 (45.7%)	95 (48.7%)	
NSTEMI (*n*, %)	6 (17.1%)	37 (19%)	
Other loc. (*n*, %)	3 (8.6%)	8 (4.1%)	
Killip I (*n*, %)	17 (48.6%)	137 (70.3%)	0.017 ^f^
Killip II (*n*, %)	13 (37.1%)	50 (25.6%)	
Killip III (*n*, %)	4 (11.4%)	7 (3.6%)	
Killip IV (*n*, %)	1 (2.9%)	1 (0.5%)	
BBB (*n*, %)	3 (8.6%)	22 (12.8%)	0.586 ^f^
VF (*n*, %)	1 (2.9%)	11 (5.6%)	0.698 ^f^
VT (*n*, %)	5 (14.3%)	20 (10.3%)	0.553 ^f^
A.fib (*n*, %)	3 (8.6%)	8 (4.1%)	0.380 ^f^
AV block gr. I (*n*, %)	4 (11.4%)	14 (7.2%)	0.490 ^f^
AV block gr. II–III (*n*, %)	4 (11.4%)	6 (3.1%)	0.048 ^f^

yrs.—years; MI—Myocardial infarction; CVD—Cardiovascular diseases; AS—Anteroseptal; IP—Inferoposterior; NSTEMI—Non ST-elevation MI; loc.—localization; BBB—Bundle Brunch Block; VF—Ventricular Fibrillation; VT—Ventricular Tachycardia; A.fib.—Atrial Fibrillation; AV—Atrioventricular; gr.—grade; SD—Standard deviation; ^t^—Independent-Samples *t* test; ^c^—Pearson Chi Square; ^f^—Fisher Exact test.

**Table 2:** Short-term time domain parameters of Heart Rate Variability at Day 1 and Day 21 after myocardial infarction and corresponding delta values of the study population.

	Died *N* = 35	Survived *N* = 195	*p* Value
**1st day**			
RR interval (ms) (mean ± SD)	757.1 ± 227.8	791.6 ± 170.5	0.298 ^t^
SDNN (ms) (Mdn (IQR))	11.5 (6.8–19.3)	10.5 (5–17.8)	0.696 ^m^
PNN50 (%) (Mdn (IQR))	1 (0–2)	1 (0–4)	0.600 ^m^
RMSSD (ms) (Mdn (IQR))	16.5 (10.8–23.5)	16 (8–28.9)	0.943 ^t^
**21st day**			
RR interval (ms) (mean ± SD)	777.6 ± 160.7	878.7 ± 155.2	0.001 ^t^
SDNN (ms) (Mdn (IQR))	6 (4–9)	10 (6–17)	0.001 ^m^
PNN50 (%) (Mdn (IQR))	0 (0–0)	0 (0–3)	0.001 ^m^
RMSSD (ms) (Mdn (IQR))	9 (6–17)	16 (9–27)	0.001 ^m^
Δvalues (after–before) *			
RR interval (ms) (Mdn)	83 (−53–139)	79 (−23–196)	0.434 ^m^
SDNN (ms) (Mdn)	−4 (−9–2)	1 (−5–5)	0.012 ^m^
PNN50 (%) (Mdn)	0 (−2–0)	0 (−2–1)	0.083 ^m^
RMSSD (ms) (Mdn)	−5 (−12–3)	1 (−7–8)	0.053 ^m^

SDNN—Standard deviation of normal intervals; PNN50—Percentage of adjacent NN intervals that differ > 50 ms; RMSSD—Root Mean Square of Successive Differences; ms—milliseconds; *—Δ values represent change from baseline (after–before); SD—Standard deviation; Mdn—Median; IQR—Interquartile range (25–75%); ^t^—Independent samples *t* test; ^m^—Mann–Whitney U test.

**Table 4:** All significant predictors in univariable and final multivariable Cox Analysis model of mortality after myocardial infarction.

	*N* (%) of Patients	Mean Survival (Months) (95 CI%)	Univariable HR (95% CI)	Multivariable HR (95% CI)
**Baseline characteristics**				
Age (yrs.)			1.048 (1.011–1.087) *	1.050 (1.012–1.089) *
**Clinical status at admission**				
Killip I	17 (11%)	55.4 (53.2–57.6)	^1^	
Killip II	13 (20.6%)	50.2 (45.4–55)	2.269 (1.099–4.684) *	
Killip III	4 (36.4%)	42 (27.6–56.2)	3.998 (1.340–11.865) *	
Killip IV	1 (50%)	48 (48–48)	4.575 (0.607–34.466)	
Without AV block gr. II–III	31 (14.1%)	53.8 (51.7–55.9)	^1^	
With AV block gr. II–III	4 (40%)	44.4 (31.6–57.2)	3.253 (1.148–9.219) *	
**Echocardiographic parameters**				
Without pericardial eff.	29 (13.6%)	53.9 (51.8–56.1)	^1^	
With pericardial eff.	6 (35.3%)	47 (37.9–56)	2.574 (1.068–6.2) *	
**In-hospital therapy**				
Without BB	13 (24.1%)	48.9 (43.4–54.3)	^1^	
With BB	22 (12.5%)	54.7 (52.6–56.9)	0.468 (0.236–0.930) *	
Without UFH/LMWH	8 (32%)	48.2 (40.6–55.8)	^1^	
With UFH/LMWH	27 (13.2%)	54.1 (51.9–56.2)	0.404 (0.183—0.888) *	
**Conventional ECG parameters**				
1st day QRS < 120 ms	29 (13.7%)	54.2 (52.1–56.2)	^1^	
1st day QRS ≥ 120 ms	6 (31.6%)	44.4 (33.7–55.1)	2.811 (1.166–6.777) *	
**Short-term time domain parameters of Heart Rate Variability**				
21st day RMSSD (ms)			0.949 (0.913–0.986) *	
21st day PNN50 (%)			0.676 (0.482–0.948) *	
21st day SDNN (ms)			0.942 (0.893–0.994) *	
21st day RR interval (ms)			0.996 (0.993–0.998) *	0.996 (0.994–0.998) *
ΔSDNN increase	14 (10.6%)	55.5 (53.2–57.9)	^1^	
ΔSDNN decrease	21 (21.4%)	50.5 (46.7–54.2)	2.125 (1.081–4.179) *	
**Short-term frequency domain parameters of Heart Rate Variability**				
21st day HF (ms^2^)			0.982 (0.968–0.997) *	
ΔHF increase	12 (10.4%)	55.3 (52.8–57.9)	^1^	
ΔHF decrease	23 (20%)	51.3 (48–54.6)	2.070 (1.029–4.162) *	
ΔLF increase	9 (9.3%)	51.5 (48.4–54.5)	^1^	
ΔLF decrease	26 (19.5%)	56 (53.3–58.6)	2.230 (1.045–4.761) *	2.361 (1.091–5.108) *

yrs.—Years; AV—Atrioventricular; gr.—grade; eff.—effusion; BB—Beta blockers; UFH—unfractioned heparin; LMWH—Low-molecular-weight heparin; HF—High Frequency; ms^2^—milliseconds squared; RMSSD—Root Mean Square of Successive Differences; PNN50—Percentage of adjacent NN intervals that differ > 50 ms; SDNN—Standard deviation of normal intervals; LF—Low Frequency; Δ—represents change from baseline (after–before); CI—Confidence interval; HR—Hazard ratio; ^1^—reference category; *—*p* values < 0.05.

## Data Availability

The original contributions presented in this study are included in the article and its Supplementary Materials. Further inquiries can be directed to the corresponding author.

## Supplementary Materials

The following supporting information can be downloaded at: https://www.mdpi.com/article/10.3390/diagnostics16060942/s1. Table S1: Echocardiographic parameters of study populations. Table S2: In-hospital medical therapy of the study population. Table S3: Conventional ECG parameters at Day 1 and Day 21 after myocardial infarction and corresponding delta values of the study population. Table S4: Baseline characteristics and clinical status upon admission of the study population based on change in ΔLF. Table S5: Echocardiographic parameters of study populations based on change in ΔLF. Table S6: In-hospital medical therapy of the study population based on change in ΔLF. Table S7: Univariable Cox regression analysis for baseline characteristics and clinical status upon admission of the study population. Table S8: Univariable Cox regression analysis for echocardiographic parameters of the study population. Table S9: Univariable Cox regression analysis for in-hospital therapy of the study population. Table S10: Univariable Cox regression analysis for conventional ECG parameters at Day 1 and Day 21 after myocardial infarction and corresponding delta values of the study population. Table S11: Univariable Cox regression analysis for short-term time domain parameters of Heart Rate Variability at Day 1 and Day 21 after myocardial infarction and corresponding delta values of the study population. Table S12: Univariable Cox regression analysis for short-term frequency domain parameters of Heart Rate Variability at Day 1 and Day 21 after myocardial infarction and corresponding delta values of the study population. Table S13: Association between beta-blocker therapy and ΔLF.
