# Supplementary material for: Short-Term Heart Rate Variability Dynamics and Mortality Risk After Acute Coronary Syndrome
Source: Diagnostics (Basel). 2026 Mar 23;16(6):942. doi: 10.3390/diagnostics16060942 (PMC13025100; doi:10.3390/diagnostics16060942)
Supplement: Supplementary file 1 [file diagnostics-16-00942-s001.zip › diagnostics-4195924-supplementary.pdf]

Supplementary Table S1. Echocardiographic parameters of study populations.

	Died N = 35	Survived N = 195	P value
EF (mean ± SD)	45.7 ± 13.6	48.3 ± 11.3	.242[t]
EF ≤ 40% (n,%)	15 (42.9%)	63 (32.3%)	.225[c]
Left atrial dilatation (n,%)	12 (34.3%)	65 (33.5%)	.928[c]
Left ventricular dilatation (n,%)	12 (34.3%)	53 (27.2%)	.390[c]
WMA (n,%)	33 (94.3%)	184 (94.4%)	1.000[f]
IVS hypertrophy (n,%)	8 (22.9%)	51 (26.2%)	.686[c]
LV hypertrophy (n,%)	5 (14.3%)	30 (15.4%)	.868[c]
Intracardiac thrombus (n,%)	5 (14.3%)	12 (6.2%)	.149[f]
Left ventricular aneurysm (n,%)	6 (17.1%)	24 (12.3%)	.420[f]
Pericardial effusion (n,%)	6 (17.1%)	11 (5.6%)	.028[f]

EF—Ejection Fraction; WMA—Wall motion abnormalities; IVS—Interventricular septum; LV—Left Ventricle; SD—Standard deviation; [t]—Independent-Samples T test; [c]—Pearson Chi Square; [f]—Fisher Exact test.

Supplementary table S2. In-hospital medical therapy of the study population.

	Died N = 35	Survived N = 195	P value
Fibrinolytic therapy (n,%)	8 (22.9%)	55 (28.2%)	.514[c]
Nitroglycerin (n,%)	32 (91.4%)	184 (94.4%)	.453[f]
Beta blockers (n,%)	22 (62.9%)	154 (79%)	.038[c]
Amiodarone (n,%)	0	13 (6.7%)	.227[f]
Digoxin (n,%)	1 (2.9%)	9 (4.6%)	1.000[f]
Diuretics (n,%)	21 (60%)	115 (59%)	1.000[f]
OAK (n,%)	5 (14.3%)	32 (16.4%)	.753[c]
DAPT (n,%)	33 (94.3%)	175 (89.7%)	.542[f]
Lidocaine (n,%)	10 (28.6%)	56 (28.7%)	.986[c]
ACE inhibitors (n,%)	26 (74.3%)	152 (77.9%)	.633[c]
UFH/LMWH (n,%)	27 (77.1%)	178 (91.3%)	.033[f]

OAK—Oral anticoagulation therapy; DAPT—Double antiplatelet therapy; ACE—Angiotensin Converting Enzyme; UFH—Unfractionated heparin; LMWH—Low-molecular-weight heparin; [c]—Pearson Chi Square; [f]—Fisher Exact test.

Supplementary table S3. Conventional ECG parameters at Day 1 and Day 21 after myocardial infarction and corresponding delta values of the study population.

	Died N = 35	Survived N = 195	P value
**1st day**			
P wave (ms) (mean ± SD)	102.9 ± 16.3	106.6 ± 17.3	.239[t]
PQ (ms) (mean ± SD)	148.7 ±16.9	152.8 ± 32.6	.474[t]
QRS (ms) (mean ± SD)	98.3 ± 18.8	94.7 ± 17.5	.266[t]
QTc (ms) (mean ± SD)	447.5 ± 38.8	441.2 ± 37.5	.364[t]
**21st day**			
P wave (ms) (mean ± SD)	110.2 ± 17.3	110 ± 18.1	.879[t]
PQ (ms) (mean ± SD)	156.1 ± 21.5	161.5 ± 31.3	.336[t]
QRS (ms) (mean ± SD)	95.1 ± 14.9	95.3 ± 17.4	.954[t]
QTc (ms) (mean ± SD)	445.8 ± 32.1	436.1 ± 32.6	.099[t]
**Δvalues (after – before)***			
P wave (ms) (Mdn(IQR))	4 (-6 - 14)	4 (-6 - 12)	.783[m]
PQ (ms) (Mdn(IQR))	8 (-6 – 18)	8 (0 – 20)	.474[m]
QRS (ms) (Mdn(IQR))	0 (-12 – 4)	2 (-4 – 6)	.299[m]
QTc (ms) (Mdn(IQR))	-4 (-37 – 32)	- 9 (-36 -26)	.644[m]

SD—Standard deviation; Mdn—Median; * Δ values represent change from baseline (after–before); [t]—Independent samples t test; [m]—Mann–Whitney U test

Supplementary table S4. Baseline characteristics and clinical status upon admission of the study population based on change in ΔLF.

	Decreased ΔLF<br>N = 133	Increased ΔLF<br>N = 97	P value
Baseline characteristics			
Age (yrs.) (mean ± SD)	58.8 ± 9	59 ± 9.1	.700[t]
Male (n,%)	87 (65.4%)	66 (68%)	.677[c]
Previous MI (n,%)	12 (9%)	9 (9.3%)	.947[c]
Hypertension (n,%)	86 (64.7%)	76 (78.4%)	.025[c]
Hyperlipoproteinemia (n,%)	87 (65.4%)	65 (67 %)	.801[c]
Active smoking (n,%)	78 (58.6%)	57 (58.8%)	.986[c]
Family history of CVD (n,%)	62 (46.6%)	49 (50.5%)	.559[c]
Diabetes Mellitus (n,%)	49 (36.8%)	34 (34%)	.659[c]
Clinical status upon admission			
AS MI (n,%)	27 (27.8%)	29 (28.9%)	.951[c]
IP MI (n,%)	43 (47.4%)	48 (49.5%)	
NSTEMI (n,%)	26 (19.5%)	17(17.5%)	
Other loc. (n,%)	7 (5.3%)	4 (4.1%)	
Killip I (n,%)	94 (70.7%)	60 (61.9%)	.313[f]
Killip II (n,%)	34 (25.6%)	29 (29.9%)	
Killip III (n,%)	4 (3%)	7 (7.2%)	
Killip IV (n,%)	1 (.8%)	1 (1%)	
BBB (n,%)	16 (12%)	12 (12.4%)	.938[c]
VF (n,%)	5 (3.8%)	7 (7.2%)	.244[c]
VT (n,%)	15 (11.3%)	10 (10.3%)	.816[c]
A.fib (n,%)	6 (4.5%)	5 (5.2%)	1.000[f]
AV block gr. I (n,%)	11 (8.3%)	7 (7.2%)	.769[c]
AV block gr. II - III (n,%)	8 (6%)	2 (2.1%)	.198[f]

LF—Low frequency; Δ—values represent change from baseline (after–before); yrs.—years; MI—Myocardial infarction; CVD—Cardiovascular diseases; AS—Anteroseptal; IP—Inferoposterior; NSTEMI—Non-ST-elevation MI; loc.—localization; BBB—Bundle Brunch Block; VF—Ventricular Fibrillation; VT—Ventricular Tachycardia; A.fib.—Atrial Fibrillation; AV—Atrioventricular; gr.—grade; SD—Standard deviation; [f]—Independent-Samples T test; [f]—Fisher Exact test; [c]—Pearson Chi Square.

Supplementary Table S5. Echocardiographic parameters of study populations based on change in ΔLF.

	Decreased ΔLF N = 133	Increased ΔLF N = 97	P value
EF (mean ± SD)	46.9 ± 11.9	49.3 ± 11.8	.133[t]
EF ≤ 40% (n,%)	51 (38.3%)	27 (27.8%)	.096[c]
Left atrial dilatation (n,%)	43 (32.6%)	34 (35.1%)	.695[c]
Left ventricular dilatation (n,%)	39 (29.3%)	26 (26.8%)	.675[c]
WMA (n,%)	124 (93.2%)	93 (95.9%)	.391[c]
IVS hypertrophy (n,%)	31 (23.3%)	28 (28.9%)	.341[c]
LV hypertrophy (n,%)	18 (13.5%)	17 (17.5%)	.405[c]
Intracardiac thrombus (n,%)	12 (9%)	5 (5.2%)	.268[c]
Left ventricular aneurysm (n,%)	21 (15.8%)	9 (9.3%)	.148[c]
Pericardial effusion (n,%)	10 (7.5%)	7 (7.2%)	.931[c]

LF—Low frequency; Δ—values represent change from baseline (after–before); EF—Ejection Fraction; WMA—Wall motion abnormalities; IVS—Interventricular septum; LV—Left Ventricle; SD—Standard deviation; [t]—Independent-Samples T test; [c]—Pearson Chi Square.

Supplementary Table S6. In-hospital medical therapy of the study population based on change in ΔLF.

	Decreased ΔLF N = 133	Increased ΔLF N = 97	P value
Fibrinolytic therapy (n,%)	30 (30.1%)	23 (23.7%)	.285[c]
Nitroglycerin (n,%)	125 (94%)	91 (93.8%)	.957[c]
Beta blockers (n,%)	100 (75.2%)	76 (78.4%)	.576[c]
Amiodarone (n,%)	7 (5.3%	6 (6.2%)	.765[c]
Digoxin (n,%)	4 (3%)	6 (6.2%)	.329[f]
Diuretics (n,%)	74 (55.6%)	62 (63.9%)	.207[c]
OAK (n,%)	23 (17.3%)	14 (14.4%)	.560[c]
DAPT (n,%)	118 (88.7%)	90 (92.8%)	.301[c]
Lidocaine (n,%)	37 (27.8%)	29 (29.9%)	.731[c]
ACE inhibitors (n,%)	98 (73.7%)	80 (82.5%)	.116[c]
UFH/LMWH (n,%)	119 (89.5%)	86 (88.7%)	.845[c]

LF—Low frequency; Δ—values represent change from baseline (after–before); OAK—Oral anticoagulation therapy; DAPT—Double antiplatelet therapy; ACE—Angiotensin Converting Enzyme; UFH—Unfractionated heparin; LMWH—Low-molecular-weight heparin; [c]—Pearson Chi Square; [f]—Fisher Exact test.

Supplementary Table S7. Univariable Cox regression analysis for baseline characteristics and clinical status upon admission of the study population.

	N (%) of patients	Mean survival (months) (95 CI%)	Univariable HR (95% CI)	P value
**Baseline characteristics**				
Age (yrs.)			1.048 (1.011-1.087)*	.010
Age (< 60 yrs)	14 (11.2%)	55 (52.3 – 57.8)	1	.072
Age (=> 60 yrs)	21 (20%)	51.4 (48 – 54.8)	1.859 (.945 – 3.657)	
Female	14 (18.2%)	51.9 (48 – 55.8)	1	.298
Male	21 (13.7%)	54 (48.3 – 55.8)	.698 (.355 – 1.373)	

Without previous MI	31 (14.8%)	53.7 (51.5 – 55.8)	1	.525
With previous MI	4 (19%)	50.2 (41.6 – 58.9)	1.402 (.495-3.971)	
Without HTA	12 (17.6%)	52.5 (48.4 – 56.7)	1	.495
With HTA	23 (14.2%)	53.7 (51.2 – 56.2)	.786 (.391 – 1.579)	
Without HLP	16 (20.5%)	51 (47 – 55.1)	1	.091
With HLP	35 (12.5%)	54.5 (52.1 – 56.9)	.563 (.290 – 1.096)	
Non smokers	18 (18.9%)	51.6 (48 – 55.2)	1	.177
Smokers	17 (12.6%)	54.6 (52.1 – 57.1)	.633 (.326 - 1.229)	
Without family history	22 (18.5%)	51 (47 – 55.1)	1	.126
With family history	13 (11.7%)	54.5 (52.1 – 57)	.585 (.295 – 1.162)	
Without DM	19 (12.8%)	54.6 (52.2 – 57)	1	.140
With DM	16 (19.5%)	51 (47 – 55)	1.652 (.849 – 3.214)	
**Clinical status at admission**				
AS MI	10 (15.4%)	53.3 (49.3- 57.4)	1	
IP MI	16 (14.4%)	53.8 (50.9 – 56.7)	.869 (.393-1.911)	.723
NSTEMI	6 (14%)	53.4 (48.5 – 58.3)	.923 (.335-2.539)	.867
Other loc.	3 (27.3%)	48.1 (36.1-60.1)	1.992 (.584-7.240)	.295
Killip I	17 (11%)	55.4 (53.2-57.6)	1	
Killip II	13 (20.6%)	50.2 (45.4-55)	2.269 (1.099-4.684)*	.027
Killip III	4 (36.4%)	42 (27.6-56.2)	3.998 (1.340-11.865)*	.013
Killip IV	1 (50%)	48 (48-48)	4.575 (.607 – 34.466)	.140
Without BBB	32 (15.8%)	53.7 (51 – 55.5)	1	.666
With BBB	3 (10.7%)	54.2 (48.1 –60.4)	.770 (.236 -2.518)	
Without VF	34 (15.6%)	53.3 (51.8 – 55.4)	1	.537
With VF	1 (8.3%)	55.4 (46.8 – 64)	.535 (.073 – 3.906)	
Without VT	30 (14.6%)	53.7 (51.5 – 55.9)	1	.374
With VT	5 (20%)	50.3 (42.8 – 57.9)	1.538 (.596 – 3.969)	
Without A. Fib.	32 (14.6%)	53.8 (51.7 – 55.9)	1	.158
With A.Fib.	3 (27.3%)	44.7 (30 – 59.5)	2.346 (.718- 7.688)	
Without AV block gr. I	31 (14.6%)	53.8 (51.7 – 55.9)	1	.235
With AV block gr. I	4 (22.2%)	48.8 (39 – 58.5)	1.882 (.663 – 5.343)	
Without AV block gr. II-III	31 (14.1%)	53.8 (51.7 – 55.9)	1	.023
With AV block gr. II-III	4 (40%)	44.4 (31.6 -57.2)	3.253 (1.148 – 9.219)*	

CI.—Confidence interval; HR—Hazard ratio; yrs.—years; MI—Myocardial Infarction; HTA—Hypertension; HLP—Hyperlipoproteinemia; DM—Diabetes Mellitus; AS—Anteroseptal; IP—Inferoposterior; NSTEMI—Non-ST-elevation myocardial infarction; loc.—localization; BBB—Bundle brunch block; VF—Ventricular fibrillation; VT—Ventricular tachycardia; A. Fib—Atrial fibrillation; AV—Atrioventricular; gr.—grade; 1—reference category; * p value < .05

Supplementary Table S8. Univariable Cox regression analysis for echocardiographic parameters of the study population.

	N (%) of patients	Mean survival (months) (95 CI%)	Univariable HR (95% CI)	P value
EF (%)			.985 (.958 – 1.013)	.288
EF > 40%	20 (13.2%)	54.3 (51.9 – 57)	[1]	.267
EF ≤ 40%	15 (19.2%)	51.6 (47.5 – 55.6)	1.462 (.748 – 2.855)	
Without LA dilatation	23 (15.1%)	53.5 (50.9 – 56)	[1]	.919
With LA dilatation	12 (15.6%)	53.1 (49.4 – 56.8)	1.037 (.516 – 2.084)	
Without LV dilatation	23 (13.9%)	54.1 (51.8 – 56.5)	[1]	.366
With LV dilatation	12 (18.5%)	51.3 (46.7 – 55.9)	1.379 (.686 – 2.773)	
Without LV hypertrophy	30 (15.4%)	53.4 (51.1 – 55.6)	[1]	.823
With LV hypertrophy	5 (14.3%)	53.3 (47.9 – 58.8)	.898 (.348 – 2.314)	
Without IVS hypertrophy	27 (15.8%)	53.4 (51 – 55.8)	[1]	.774
With IVS hypertrophy	8 (13.6%)	53.1 (48.6 – 57.5)	.891 (.404 – 1.963)	
Without WMA	2 (15.4%)	55.1 (48 - 62.1)	[1]	.991
With WMA	33 (15.2%)	53.3 (51.1 – 55.5)	.992 (.238 – 4.133)	
Without intr. thrombus	30 (14.1%)	53.7 (51.5 – 55.8)	[1]	.090
With intr. Thrombus	5 (29.4%)	50.2 (41.8 – 58.9)	2.266 (.879 - 5.841)	
Without LV aneurysm	29 (14.5%)	53.4 (51.1 – 55.7)	[1]	.601
With LV aneurysm	6 (20%)	53.4 (47.8 – 58.9)	1.264 (.525 – 3.046)	
Without PE	29 (13.6%)	54 (51.8 – 56.1)	[1]	.035
With PE	6 (35.3%)	47 (37.9 – 56)	2.574 (1.068 – 6.200)*	

CI—Confidence interval; HR—Hazard ratio; EF—Ejection fraction; LA—Left atrium; LV—Left ventricle; IVS—Interventricular septum; WMA—Wall motion abnormalities; Intr.—intracardiac; PE—Pericardial effusions; [1]—reference category; * $p < .05$.

Supplementary Table S9. Univariable Cox regression analysis for in-hospital therapy of the study population.

	N (%) of patients	Mean survival (months) (95 CI%)	Univariable HR (95% CI)	P value
Not treated with fibrinolyt.	27 (16.2%)	53.1 (50.5 – 55.6)	[1]	.514
Treated with fibrinolyt.	8 (12.7%)	54.2 (50.4 – 58)	1.300 (.591 – 2.862)	
Not treated with Ntg.	3 (21.4%)	51.6 (42.9 – 60.2)	[1]	.663
Treated with Ntg.	32 (14.8%)	53.5 (51.3 – 55.7)	.769 (.235 – 2.511)	
Not treated with BB	13 (24.1%)	48.9 (43.4 – 54.3)	[1]	.030
Treated with BB	22 (12.5%)	54.7 (52.6 – 56.9)	.468 (.236 – .930)*	
Not treated with Amiodarone#	35 (16.1%)			
Treated with Amiodarone	0			
Not treated with Digoxin	34 (15.5%)	53.2 (51.1 – 55.4)	[1]	.609
Treated with Digoxin	1 (10%)	56 (48.6 – 63.4)	.595 (0.81 – 4.350)	
Not treated with Diuretics	14 (14.9%)	54.1 (51 – 57.2)	[1]	.600
Treated with Diuretics	21 (15.4%)	52.8 (49.9 – 55.7)	1.119 (.609 – 2.363)	
Not treated with OAK	30 (15.5%)	53.3 (50.8 – 55.5)	[1]	.714
Treated with OAK	5 (13.5%)	54.5 (49.6 – 59.4)	.838 (.325 – 2.159)	
Not treated with DAPT	2 (9.1%)	55.2 (48.9 – 61.6)	[1]	.377
Treated with DAPT	33 (15.9%)	53.1 (50.9 – 55.4)	1.903 (.456 – 7.937)	
Not treated with Lidocaine	25 (15.2%)	53.8 (51.4 – 56.1)	[1]	.763
Treated with Lidocaine	10 (15.2%)	52.5 (48.2 – 56.9)	1.120 (.537 – 2.334)	
Not treated with ACE inhib.	9 (17.3%)	52 (47 – 57)	[1]	.605
Treated with ACE inhib.	26 (14.6)	53.8 (51.5 – 56)	.818 (.383 – 1.747)	
Not treated UFH/LMWH	8 (32%)	48.2 (40.6 – 55.8)	[1]	.024
Treated with UFH/LMWH	27 (13.2%)	54.1 (51.9 – 56.2)	.404 (.183 - .888)*	

CI—Confidence interval; HR—Hazard ratio; fibrinolyt.—fibrinolytic; Ntg—Nitroglycerin; BB—Beta blockers; # Amiodarone was not analyzed in univariable Cox regression because in the group of patients treated with amiodarone, no one had the outcome of interest; OAK—Oral anticoagulation therapy; DAPT—Dual Antiplatelet Therapy; ACE Inihib.—Angiotensin Converting Enzyme inhibitors; UFH—Unfractionated heparin; LWMH—Low-molecular-weight heparin; [1]—reference category; * $p < .05$.

Supplementary Table S10. Univariable Cox regression analysis for **c**onventional ECG parameters at Day 1 and Day 21 after myocardial infarction and corresponding delta values of the study population.

	N (%) of patients	Mean survival (months) (95 CI%)	Univariable HR (95% CI)	P value
**1st day**				
P wave (ms)			.990 (.970 – 1.010)	.310
P wave < 120 ms	31 (16.2%)	53.2 (50.1 – 55.5)	1	.469
P wave ≥ 120 ms	4 (10.3%)	54.3 (49.1 – 59.6)	.680 (.240 – 1.928)	
PQ (ms)			.996 (.984 – 1.008)	.516
PQ < 200 ms#	35 (15.6%)			
PQ ≥ 200 ms	0			
QRS (ms)			1.010 (.995 – 1.025)	.210
QRS < 120 ms	29 (13.7%)	54.2 (52.1 – 56.2)	1	.021
QRS ≥ 120 ms	16 (31.6%)	44.4 (33.7 – 55.1)	2.811 (1.166 – 6.777)*	
QTc (ms)			1.005 (.996 – 1.013)	.275
QTc < 440/460 ms&	19 (14%)	54.4 (51.9 – 56.8)	1	.426
QTc ≥ 440/460 ms	16 (17%)	52 (48.3 – 55.7)	1.310 (.673 – 2.548)	
**21st day**				
P wave (ms)			1.002 (.984 – 1.021)	.803
P wave < 120 ms	27 (14.9%)	53.6 (51.1 – 56)	1	.774
P wave ≥ 120 ms	8 (16.3%)	52.4 (47.4 – 57.3)	1.123 (.510 – 2.472)	
PQ (ms)			.994 (.981 – 1.007)	.382
PQ < 200 ms	32 (15%)	53.3 (51.4 – 55.7)	1	.597
PQ ≥ 200 ms	3 (18.8%)	51.1 (41.9 – 60.2)	1.377 (.421 – 4.499)	
QRS (ms)			1.001 (.982 – 1.019)	.949
QRS < 120 ms	31 (14.5%)	53.8 (51.7 – 55.9)	1	.187
QRS ≥ 120 ms	4 (25%)	47.5 (36.5 – 58.5)	2.018 (.712 – 5.717)	
QTc (ms)			1.008 (.998 – 1.018)	.103
QTc < 440/460 ms	19 (13.3%)	54.3 (51.8 – 56.7)	1	.308
QTc ≥ 440/460 ms	16 (18.4%)	51.9 (48.1 – 55.7)	1.413 (.727 – 2.749)	
**Δvalues (after – before)$**				
ΔP (ms)			1.007 (.992 – 1.023)	.330
ΔP decrease	13 (16.5%)	52.8 (49 – 56.5)	1	.738
ΔP increase	22 (14.6%)	53.7 (51.1 – 56.2)	.889 (.448 – 1.776)	
ΔPQ (ms)			.999 (.993 – 1.005)	.697
ΔPQ decrease	12 (21.2%)	51.1 (46.4 – 55.9)	1	.175
ΔPQ increase	23 (13.3%)	54.1 (51.8 – 56.4)	.617 (.307 – 1.240)	
ΔQRS (ms)			.989 (.972 – 1.006)	.219
ΔQRS decrease	15 (16.9%)	52.4 (48.7 – 56)	1	.649
ΔQRS increase	20 (14.2%)	54 (51.4 – 56.5)	.856 (.438 – 1.672)	
ΔQTc (ms)			1.001 (.994 – 1.009)	.772
ΔQTc decrease	20 (14.7%)	53.4 (50.7 – 56.2)	1	.852
ΔQTc increase	15 (16%)	53.3 (50 – 56.5)	1.066 (.546 – 2.082)	

CI—Confidence interval; HR—Hazard ratio; # PQ on the 1st day, as a categoric variable, was not used in the univariable model, because one of the group did not have the outcome of interest; & criteria for prolonged QTc was in aligment with recommendations based on gender (male ≥440ms; female ≥ 460ms); $ Δ values represent change from baseline (after–before); 1—reference category; * $p < .05$.

Supplementary Table S11. Univariable Cox regression analysis for short-term time domain parameters of Heart Rate Variability at Day 1 and Day 21 after myocardial infarction and corresponding delta values of the study population.

	N (%) of patients	Mean survival (months) (95 CI%)	Univariable HR (95% CI)	P value
**1st day**				
RR interval (ms)			.999 (.997 – 1.001)	.249
RR interval > 760 ms	14 (12.2%)	54.5 (51.7 - 57.2)	1	.204
RR interval ≤ 760 ms	21 (18.3%)	52.2 (49 – 55.4)	1.551 (.789 – 3.050)	
SDNN (ms)			.994 (.975 – 1.013)	.531
SDNN > 10 ms	18 (15.8%)	53 (50 – 56.1)	1	.848
SDNN ≤ 10ms	17 (14.7%)	53.9 (50.8 – 56.6)	.937 (.483 – 1.819)	
PNN50 (%)			.943 (.872 – 1.020)	.142
PNN50 ≥ 2%	11 (12.8%)	54.4 (51.3 – 57.6)	1	.414
PNN50 < 2%	24 (16.7%)	52.7 (49.9 – 55.5)	1.347 (.660 – 2.749)	
RMSSD (ms)			.990 (.973 – 1.008)	.268
RMSSD > 16ms	17 (15.5%)	52.9 (49.8 – 56.1)	1	.909
RMSSD ≤ 16ms	18 (15%)	53.8 (51 – 56.5)	.962 (.496 – 1.867)	
**21st day**				
RR interval (ms)			.996 (.993 – .998)*	< .001
RR interval > 850 ms	9 (7.8%)	56.6 (54.3 – 58.8)	1	.003
RR interval ≤ 850 ms	26 (22.6%)	50.1 (46.6 – 53.6)	3.179 (1.489 – 6.786)	
SDNN (ms)			.942 (893 – .994)*	029
SDNN > 10 ms	8 (8.2%)	56 (53.3 – 58.7)	1	.016
SDNN ≤ 10ms	27 (20.5%)	51.4 (48.4 – 54.5)	2.640 (1.119 – 5.811)	
PNN50 (%)			.676 (.482 - .948)*	.023
PNN50 ≥ 2%	3 (4.4%)	57.8 (55.8 – 60.2)	1	.008
PNN50 < 2%	32 (19.8%	51.4 (48.6 – 54.2)	4.972 (1.522 – 16.242)	
RMSSD (ms)			.949 (.913 – .986)*	.008
RMSSD > 15 ms	11 (10.1%)	55.1 (52.3 – 57.9)	1	.050
RMSSD ≤ 15 ms	24 (19.8%)	51.8 (48.7 – 54.9)	2.043 (1.000 – 4.170)	
**Δvalues (after – before)$**				
ΔRR (ms)			.999 (.997 – 1.000)	.092
ΔRR increase	12 (17.6%)	52 (47.8 – 56.2)	1	.545
ΔRR decrease	23 (14.2%)	53.9 (51.4 – 56.3)	1.241 (.617 – 2.494)	
ΔSDNN (ms)			.997 (.986 – 1.008)	.565
ΔSDNN increase	14 (10.6%)	55.5 (53.2 – 57.9)	1	.029
ΔSDNN decrease	21 (21.4%)	50.4 (46.7 – 54.2)	2.215 (1.081 – 4.179)	
ΔPNN50 (%)			1.002 (.974 – 1.030)	.896
ΔPNN50 increase	19 (12.9%)	54.4 (51.9 – 55.5)	1	.232
ΔPNN50 decrease	16 (19.3%)	51.6 (47.7 – 55.5)	1.501 (.772 – 2.918)	
ΔRMSSD (ms)			.999 (.991 – 1.006)	.766
ΔRMSSD increase	15 (12.2%)	54.8 (52.1 – 57.4)	1	.179
ΔRMSSD decrease	20 (18.7%)	51.8 (48.4 – 55.1)	1.583 (.810 – 3.092)	

CI—Confidence interval; HR—Hazard ratio; SDNN—Standard deviation of normal intervals; PNN50—Percentage of adjacent NN intervals that differ > 50ms; RMSSD—Root Mean Square of Successive Differences; ms—milliseconds; $ Δ values represent change from baseline (after–before); 1—reference category; * p < .05.

Supplementary Table S12. Univariable Cox regression analysis for short-term frequency domain parameters of Heart Rate Variability at Day 1 and Day 21 after myocardial infarction and corresponding delta values of the study population.

	N (%) of patients	Mean survival (months) (95 CI%)	Univariable HR (95% CI)	P value
**1st day**				
VLF (ms²)			1.000 (.999 – 1.001)	.825
VLF > 72 ms²	14 (12.3%)	54.3 (51.4 - 57.2)	1	.233
VLF ≤ 72 ms²	21 (18.1%)	52.5 (49.4 – 55.5)	.662 (.337 – 1.303)	
LF (ms²)			1.000 (.999 – 1.001)	.676
LF > 50 ms²	18 (15.8%)	52.7 (49.6 – 56.9)	1	.851
LF ≤ 50 ms²	17 (14.7%)	54 (51.2 – 56.7)	.938 (.484 – 1.821)	
HF (ms²)			.999 (.996 – 1.002)	.411
HF > 21 ms²	19 (18.6%)	52.8 (49.8 – 55.9)	1	.597
HF ≤ 21 ms²	16 (13.7%)	54 (51.1 – 56.8)	.836 (.430 – 1.625)	
LF/HF			1.014 (.972 – 1.058)	.524
LF/HF 1.5 - 2	2 (8.3%)	56.5 (51.9 – 61.2)	1	
LF/HF < 1.5	12 (14.6%)	53.9 (50.5 – 57.3)	1.889 (.423 – 8.443)	.405
LF/HF > 2	21 (16.9%)	52.4 (49.3 – 55.5)	2.248 (.527 - 9.590)	.274
**21st day**				
VLF (ms²)			.996 (.991 – 1.000)	.057
VLF > 62 ms²	15 (13.2%)	53.8 (50.8 – 56.8)	1	.460
VLF ≤ 62 ms²	20 (17.2%)	53 (50 – 56)	1.287 (.659 – 2.514)	
LF (ms²)			.994 (.987 – 1.000)	.056
LF > 40 ms²	13 (11.6%)	54.5 (51.7 – 57.4)	1	.151
LF ≤ 40 ms²	22 (18.6%)	52.2 (49.2 – 55.3)	1.653 (.833 – 3.282)	
HF (ms²)			.982 (.968 - .997)*	.017
HF > 21 ms²	10 (8.8%)	55.9 (53.4 – 58.4)	1	.009
HF ≤ 21 ms²	25 (21.6%)	50.8 (47.5 – 54.2)	2.642 (1.269 – 5.504)	
LF/HF			1.053 (.976 – 1.135)	.181
LF/HF 1.5 - 2	2 (6.9%)	57.5 (53.7 – 61.3)	1	
LF/HF < 1.5	16 (15.7%)	53 (49.7 – 56.2)	2.610 (.600 – 11.363)	.201
LF/HF > 2	17 (17.2%)	52.5 (49.2 – 55.9)	2.937 (.678 – 12.276)	.150
**Δvalues (after – before)[$]**				
ΔVLF (ms²)			1.000 (.999 – 1.001)	.599
ΔVLF increase	15 (13.4%)	54.4 (51.7 – 57.2)	1	.384
ΔVLF decrease	20 (17.4%)	52.3 (51.7 – 57.2)	1.346 (.689 – 2.630)	
ΔLF (ms²)			1.000 (.999 – 1.001)	.990
ΔLF increase	9 (9.3%)	56 (53.3 – 58.6)	1	.038
ΔLF decrease	26 (19.5%)	51.5 (48.4 – 54.5)	2.230 (1.045 – 4.761)	
ΔHF (ms²)			1.000 (.999 – 1.001)	.655
ΔHF increase	12 (10.4%)	53.3 (52.8 – 57.9)	1	.041
ΔHF decrease	23 (20%)	51.3 (48 – 54.6)	2.070 (1.029– 4.162)	
ΔLF/HF			1.003 (.948 – 1.061)	.918
ΔLF/HF increase	16 (16.2%)	52.9 (49.6 – 56.4)	1	.701
ΔLF/HF decrease	19 (14.5%)	53.7 (51 – 56.4)	.878 (.451 – 1.707)	

CI—Confidence interval; HR—Hazard ratio; VLF—Very Low Frequency; LF—Low Frequency; HF—High Frequency; ms^2—milliseconds squared; ms^2—milliseconds squared; [$] Δ values represent change from baseline (after–before); [1]—reference category; * $p < .05$.

Supplementary Table S13. Association between beta-blocker therapy and ΔLF.

Analysis	Predictor	Effect estimate	p value	95% Confidence Interval
**Logistic regression[1]**	Beta-blocker therapy	OR = 1.19	0.577	0.64 - 2.23
**Linear regression[2]**	Beta-blocker therapy	B = −24.81	0.773	-194.37 - 144.75

[1] **Logistic regression model**: Dependent variable: ΔLF categorized as increase vs. decrease.
Predictor: beta-blocker therapy (yes/no).
Odds ratios (OR) with 95% confidence intervals are reported.
Model statistics: Wald $\chi^2 = 0.312$, $p = 0.577$.
Model fit: −2 log likelihood = 312.876; Cox–Snell $R^2 = 0.001$; Nagelkerke $R^2 = 0.002$.

[2] **Linear regression model**: Dependent variable: ΔLF analyzed as a continuous variable.
Predictor: beta-blocker therapy (yes/no).
Unstandardized regression coefficient (B) with 95% confidence interval is reported.
Test statistic: $t = -0.288$, $p = 0.773$.
Model statistics: $F = 0.083$, $p = 0.773$; $R^2 < 0.001$.
